# Chemerin Isoform-Specific Effects on Hepatocyte Migration and Immune Cell Inflammation

**DOI:** 10.3390/ijms21197205

**Published:** 2020-09-29

**Authors:** Susanne Feder, Astrid Bruckmann, Nichole McMullen, Christopher J. Sinal, Christa Buechler

**Affiliations:** 1 Department of Internal Medicine I, Regensburg University Hospital, 93053 Regensburg, Germany; feder.susanne@gmx.de; 2 Biochemistry Center Regensburg (BZR), Laboratory for RNA Biology, University of Regensburg, 93042 Regensburg, Germany; astrid.bruckmann@vkl.uni-regensburg.de; 3 Department of Pharmacology, Dalhousie University, Halifax, NS B3H 4R2, Canada; Nichole.McMullen@Dal.Ca (N.M.); Christopher.Sinal@Dal.Ca (C.J.S.)

**Keywords:** GPR1, CMKLR1, Tango assay, proliferation

## Abstract

Murine chemerin is C-terminally processed to the bioactive isoforms, muChem-156 and muChem-155, among which the longer variant protects from hepatocellular carcinoma (HCC). However, the role of muChem-155 is mostly unknown. Here, we aimed to compare the effects of these isoforms on the proliferation, migration and the secretome of the human hepatocyte cell lines HepG2 and Huh7 and the murine Hepa1-6 cell line. Therefore, huChem-157 and -156 were overexpressed in the human cells, and the respective murine variants, muChem-156 and -155, in the murine hepatocytes. Both chemerin isoforms produced by HepG2 and Hepa1-6 cells activated the chemerin receptors chemokine-like receptor 1 (CMKLR1) and G protein-coupled receptor 1 (GPR1). HuChem-157 was the active isoform in the Huh7 cell culture medium. The potencies of muChem-155 and muChem-156 to activate human GPR1 and mouse CMKLR1 were equivalent. Human CMKLR1 was most responsive to muChem-156. Chemerin variants showed no effect on cell viability and proliferation. Activation of the mitogen-activated protein kinases Erk1/2 and p38, and protein levels of the epithelial–mesenchymal transition marker, E-cadherin, were not regulated by the chemerin variants. Migration was reduced in HepG2 and Hepa1-6 cells by the longer isoform. Protective effects of chemerin in HCC include the modulation of cytokines but huChem-156 and huChem-157 overexpression did not change IL-8, CCL20 or osteopontin in the hepatocytes. The conditioned medium of the transfected hepatocytes failed to alter these soluble factors in the cell culture medium of peripheral blood mononuclear cells (PBMCs). Interestingly, the cell culture medium of Huh7 cells producing the inactive variant huChem-155 reduced CCL2 and IL-8 in PBMCs. To sum up, huChem-157 and muChem-156 inhibited hepatocyte migration and may protect from HCC metastasis. HuChem-155 was the only human isoform exerting anti-inflammatory effects on immune cells.

## 1. Introduction

The multifunctional chemokine chemerin acts through the G protein-coupled receptors chemokine-like receptor 1 (CMKLR1) and G protein-coupled receptor 1 (GPR1). Chemerin’s role as an attractant for immune cells is very well described [1,2,3]. More recent studies have identified potential roles for chemerin in different cancers [4,5]. For example, chemerin expression was mainly downregulated in cancer tissues, including hepatocellular carcinoma (HCC) [4,5,6,7,8] and a protective effect of chemerin was reported in experimental HCC models [6,8,9].

Chemerin is present in human serum/plasma mainly as an inactive 143-amino acid protein. This variant is referred to as chemerin 163 in the literature although the 20 N-terminal amino acids were removed. Prochemerin (huChem-163) can be activated by C-terminal proteolytic processing [1,10,11]. The human variants huChem-156, 157 and 158 are biologically active isoforms, while those with 155 or less amino acids are generally regarded as inactive [1]. Initially, chemerin was studied mainly for its role as a chemoattractant. HuChem-157 was the most potent isoform in chemotaxis assays, and accordingly, is the most intensively studied variant so far [1,4]. The respective murine chemerin isoforms are one amino acid shorter than their human equivalents, with murine muChem-156 equivalent to huChem-157 [1].

In human hepatocyte cell lines, huChem-157 enhanced the expression and activity of phosphatase and tensin homolog (PTEN) and thereby decreased levels of phosphorylated Akt. Consequently, migration and invasion of HCC cells were suppressed [6]. However, HuChem-157 did not inhibit cell proliferation of different human HCC cell lines and, similarly, muChem-156 did not reduce the growth of murine Hepa1-6 cells [6,8]. MuChem-156-overexpressing Hepa1-6 cells had reduced nuclear factor-κB activation and granulocyte-macrophage colony-stimulating factor (GM-CSF) production [8]. In a murine diethylnitrosamine (DEN)-induced liver cancer model, muChem-156 overexpression reduced the number of early liver lesions [9]. However, larger tumors did not differ among the control and muChem-156 group [9]. Moreover, muChem-155 was the prominent active chemerin isoform in mouse liver tumors [9]. MuChem-156 and muChem-155 had a comparable activity in Ca^2+^ mobilization and chemotaxis assays [12]. This is in contrast to human chemerin, where huChem-157 was by far the most active isoform [3,13,14].

The HCC-inhibitory effects of chemerin included a shift from a tumor-supportive to a cancer-fighting immune environment [8]. CMKLR1 is expressed by T cells, monocytes/macrophages, dendritic cells, natural killer cells as well as neutrophils, and chemerin functions as a chemoattractant for all of these immune cells [15]. Moreover, huChem-157 shifted the macrophages from a pro-inflammatory M1 to an anti-inflammatory M2 phenotype [16]. In contrast, a separate study showed that muChem-156 suppressed M2 polarization of murine macrophages [17]. Direct anti-inflammatory effects of huChem-157/muChem-156 on human and murine macrophages were not identified in a third investigation [18]. Thus, the role of chemerin in inflammation remains unclear, as both pro- and anti-inflammatory effects have been described in different models [1]. Expression of the chemerin receptors GPR1 and CMKLR1 or further processing of chemerin may have a role herein. GPR1 and CMKLR1 are G protein-coupled receptors. Both receptors activate beta-arrestin 1 and 2 upon chemerin binding [19]. Several downstream signals of CMKLR1 have been identified including Akt, p38, Erk1/2, rho-associated protein kinase (Rock), Src and protein kinase C [20,21]. By comparison, signaling through GPR1 is much less well understood. The p38 kinase and Rock are downstream of GPR1 and activate serum response factor-dependent gene expression and migration [21]. While calcium mobilization and Erk1/2 phosphorylation are associated with chemerin activation of GPR1, the magnitude of response was lower compared to that mediated by CMKLR1 [19].

The aim of the current study was to analyze the role of huChem-156/muChem-155 on the proliferation, migration and secretome of human and murine hepatocyte cell lines. Hepatocyte–macrophage crosstalk has a central role in HCC [22] and the effect of hepatocyte-produced chemerin on the secretome of peripheral blood mononuclear cells was also investigated.

## 2. Results

### 2.1. Overexpression of Chemerin Isoforms in Hepa1-6 Cells

**Figure 1:**
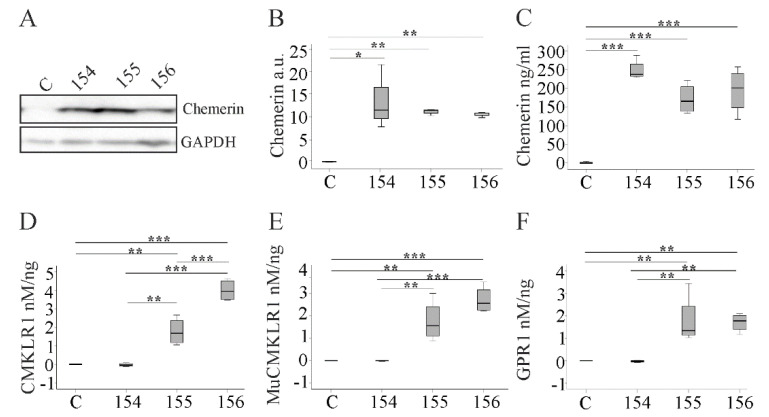
Expression of chemerin isoforms in Hepa1-6 cells. (**A**) Immunoblot of chemerin in lysates of Hepa1-6 cells expressing muChem-154, -155 or -156. C indicates Hepa1-6 cells transfected with the insertless plasmid. (**B**) Densitometry analysis of cellular chemerin measured by Western blot (arbitrary units, a. u.). (**C**) Quantification of chemerin in the media of Hepa1-6 cells by ELISA. Bioactivity of the secreted murine chemerin isoforms, corrected for total media chemerin levels, as measured by the (**D**) human CMKLR1, (**E**) murine (Mu) CMKLR1 and (**F**) human GPR1 Tango assay. Data were analyzed with one-way ANOVA with post-hoc Tukey test * *p* < 0.05, ** *p* < 0.01, *** *p* < 0.001. *n* = 4.

Hepa1-6 cells were transfected with the different recombinant plasmids to overexpress chemerin isoforms. MuChem-154 is an inactive chemerin isoform, whereas muChem-155 and -156 are both biologically active variants [1]. Endogenous chemerin was barely detectable in cell lysates of Hepa1-6 cells transfected with the plasmid without insert (Figure 1A). MuChem-154, -155 and -156 were analyzed in the cell lysates by immunoblot using a chemerin antibody, which reacted with all isoforms. As expected, expression of chemerin protein was elevated in lysates of the cells transfected with recombinant plasmids (Figure 1A,B). Secreted chemerin in the cell media was quantitated using an ELISA capable of detecting all of the recombinant isoforms. Secreted chemerin levels were similar in Hepa1-6 cells expressing muChem-154, -155 or -156 (Figure 1C).

The Tango assay can measure chemerin bioactivity using chemerin-induced interaction of the receptor with beta-arrestin 2 as a marker [1,23]. The human CMKLR1-based Tango assay indicated that muChem-155 and -156 were the active isoforms, with muChem-156 the most active overall (Figure 1D). In the murine CMKLR1 Tango assay, both isoforms showed comparable receptor activation (Figure 1E). MuChem-155 and -156 were equally active in the human GPR1 Tango assay (Figure 1F). Regardless of the receptor, endogenous chemerin bioactivity levels were very low and as expected, receptor activation by muChem-154 was also minimal.

### 2.2. Overexpression of Chemerin Isoforms in HepG2 and Huh7 Cells

**Figure 2:**
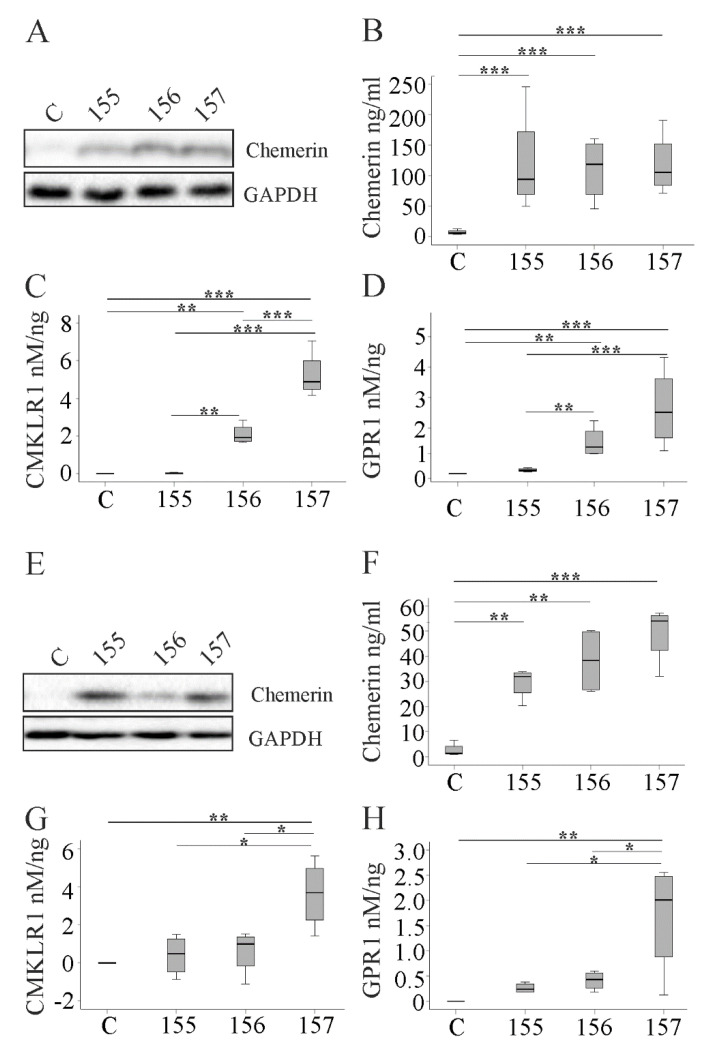
Expression of chemerin isoforms in HepG2 and Huh7 cells. (**A**) Immunoblot of chemerin in the cell lysate of HepG2 cells expressing huChem-155, -156 or -157. C indicates HepG2 cells transfected with the insertless plasmid. (**B**) Quantification of secreted chemerin in the media of HepG2 cells by ELISA. Activation of (**C**) human CMKLR1 or (**D**) human GPR1 by the human chemerin isoforms relative to total HepG2 media chemerin levels. (**E**) Immunoblot of chemerin in the cell lysate of Huh7 cells expressing huChem-155, -156 or -157. C indicates Huh7 cells transfected with the insertless plasmid. (**F**) Quantification of secreted chemerin in the media of Huh7 cells by ELISA. Activation of (**G**) human CMKLR1 or (**H**) human GPR1 by the chemerin isoforms relative to total Huh7 media chemerin levels. Data were analyzed with one-way ANOVA with post-hoc Tukey test. * *p* < 0.05; ** *p* < 0.01; *** *p* < 0.001; *n* = 4.

HepG2 cells transfected with plasmids to express huChem-155 (an inactive isoform), -156 or -157 had a higher amount of cellular and secreted chemerin, with no differences between the isoforms (Figure 2A,B). In human CMKLR1 and GPR1 Tango assays, huChem-157 was more active than huChem-156, and this difference was significant for CMKLR1 activation (Figure 2C,D).

Huh7 cells expressed all recombinant chemerin isoforms to a similar degree (Figure 2E,F). HuChem-157 activated CMKLR1 and GPR1 (Figure 2G,H). Activation of CMKLR1 (*p* = 0.343, Mann–Whitney U test) and GPR1 (*p* = 0.114, Mann–Whitney U test) by huChem-157 was comparable in Huh7 and HepG2 cells. In contrast to HepG2 cells, huChem-156 produced by Huh7 cells did not significantly activate these receptors (*p* = 0.029, for comparison of CMKLR1 and GPR1 activation by huChem-156 in HepG2 and Huh7 cells, Mann–Whitney U test) (Figure 2G,H).

HuChem-155 expressed in HepG2 or Huh7 cells did not activate CMKLR1 or GPR1 signaling. Activation of chemerin receptors was not observed when medium from control transfected cells was examined (Figure 2C,D,G,H).

### 2.3. Mass Spectrometric Analysis of Chemerin Isoforms

**Figure 3:**
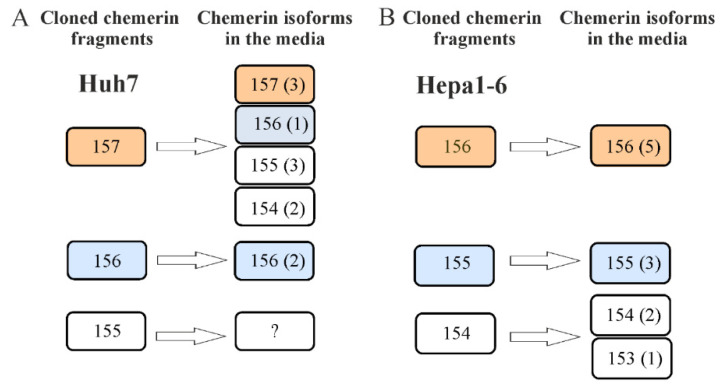
Mass spectrometric analysis of chemerin isoforms in media of Huh7 and Hepa1-6 cells. (**A**) Cloned fragments and isoforms detected by mass spectrometry in media of Huh7 cells. Numbers in brackets indicate how often the respective isoform was detected. (**B**) Cloned fragments and isoforms detected by mass spectrometry in media of Hepa1-6 cells. Number in brackets indicate how often the respective isoform was detected. *n* = 1. Bioactive isoforms are in colored boxes and inactive isoforms in colorless boxes.

Mass spectrometric analysis of chemerin in cell culture media revealed that in Huh7 cells expressing huChem-157, the isoforms huChem-157, 156, 155 and 154 were abundant in the cell culture media. HuChem-156 was the only isoform in the Huh7 cells transfected with the corresponding vector to express this isoform. Unfortunately, we failed to detect a chemerin fragment in huChem-155-expressing Huh7 cells (Figure 3A). MuChem-156 and muChem-155 were not further processed by Hepa1-6 cells. Cells expressing muChem-154 also produced muChem-153 (Figure 3B).

### 2.4. Proliferation of Chemerin-Overexpressing Hepatocytes

**Figure 4:**
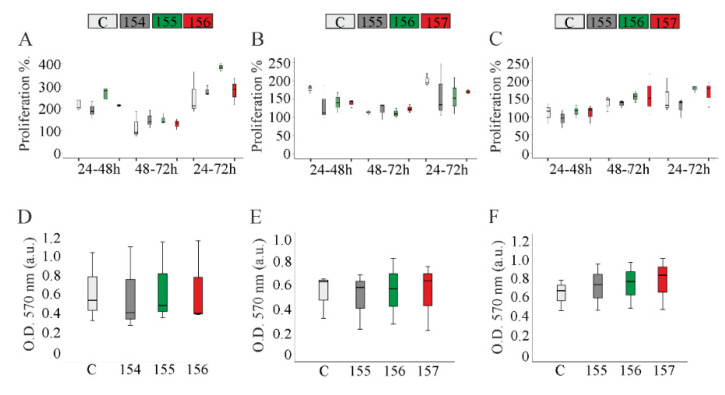
Chemerin does not affect proliferation and viability of hepatocyte cell lines. Proliferation of Hepa1-6, HepG2 and Huh7 cells. (**A**) Hepa1-6, (**B**) HepG2 or (**C**) Huh7 cells were counted 24, 48 and 72 h after transfection and the results are shown as a % of the initial cell number. MTT assay of (**D**) Hepa1-6 cells, (**E**) HepG2 cells and (**F**) Huh7 cells 48 h after transfection. Data were analyzed with one-way ANOVA with post-hoc Tukey test or the Welch test with post-hoc Games–Howell test. *n* = 3.

Previous studies reported that neither huChem-157, nor muChem-156, affected the hepatocyte proliferation [6,8]. Consistent with this, overexpression of these isoforms in Hepa1-6, HepG2 or Huh7 cells had no effect on the proliferation of these cell lines (Figure 4A–C). Overexpression of other murine (muChem-154, -155) or human (huChem-155, -156) isoforms was similarly without effect on cell proliferation (Figure 4A–C). MTT assay revealed comparable results (Figure 4D–F). Cell viability was estimated by lactate dehydrogenase determination in the cell culture media. Levels of this enzyme were not changed by any chemerin isoform (Supplementary Table S1).

### 2.5. Migration of Chemerin Isoform-Overexpressing Hepatocytes

**Figure 5:**
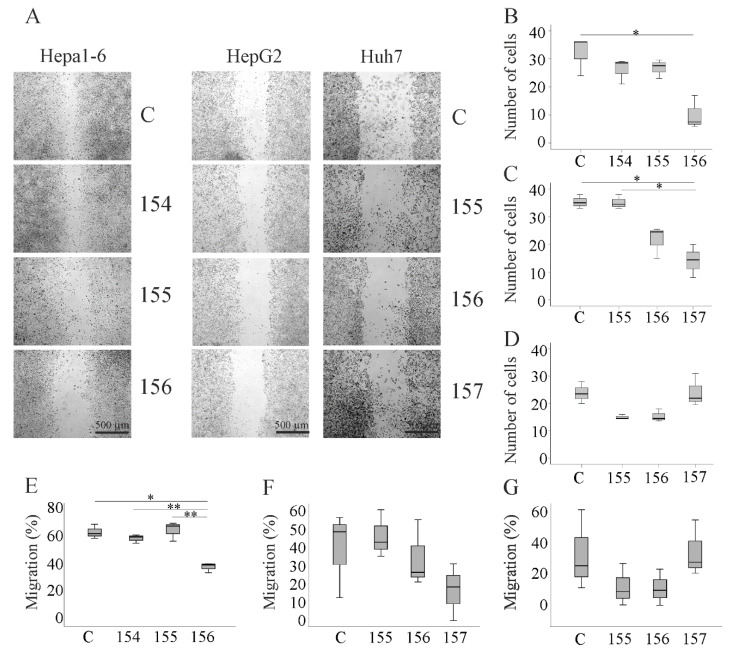
Migration of chemerin isoform-overexpressing Hepa1-6, HepG2 and Huh7 cells. (**A**) Images of the cells at 72 h (Hepa1-6, HepG2) or 48 h (Huh7) after scratching. Number of migrated (**B**) Hepa1-6, (**C**) HepG2 or (**D**) Huh7 cells expressing human chemerin isoforms at 48 h (Huh7) or 72 h (Hepa1-6, HepG2) after scratching. Data were analyzed with one-way ANOVA with post-hoc Tukey test. C are cells transfected with the insertless plasmid. % migration determined by analysis of wound healing in (**E**) Hepa1-6 and (**F**) HepG2 cells at 72 h post-transfection or (**G**) Huh7 cells at 48 h post-transfection. * *p* < 0.05; ** *p* < 0.01; *n* = 3. The statistical test used was the Welch test with post-hoc Games–Howell test.

The scratch assay was used to quantify cellular migration. Number of migrated cells and wound closure were determined. Overexpression of muChem-156 in Hepa1-6 cells inhibited cell migration and wound healing while the other two chemerin isoforms had no effect (Figure 5A,B,E). Likewise, only huChem-157 (the human equivalent of muChem-156) reduced migration of HepG2 cells (Figure 5A,C,F). Of note, none of the chemerin isoforms impaired cell migration and wound closure of Huh7 cells (Figure 5A,D,G). In some experiments, Huh7 cells detached from the cell culture plates at 72 h post-transfection. Therefore, analysis of the scratch and migrating cells was performed at 48 h post-transfection. This is a limitation of the experiments regarding Huh7 cells. However, in the two technical replicates of one experiment where cells were attached to the plate until 72 h post-transfection, there was no effect of any isoform on cell migration.

### 2.6. Activation of Extracellular Signal-Regulated Kinase (ERK)1/2 and p38 Mitogen-Activated Protein Kinase (MAPK,) and Protein Levels of E-Cadherin in Hepatocytes Overexpressing Chemerin Isoforms

**Figure 6:**
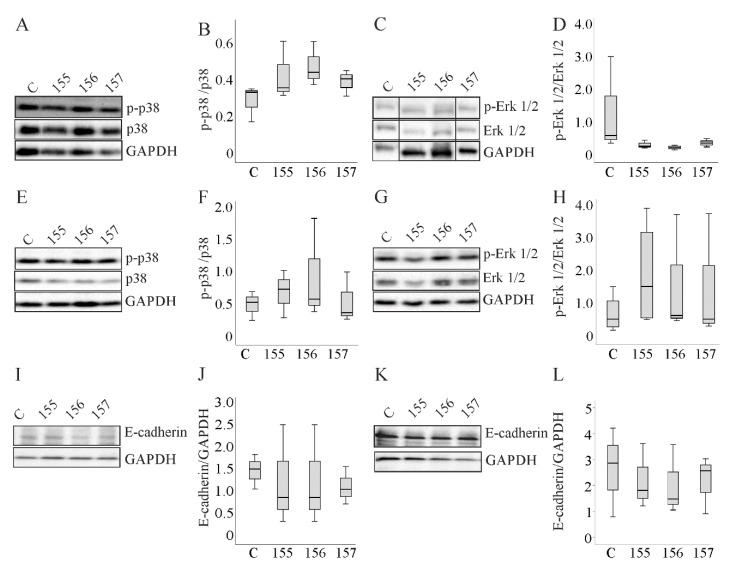
Expression of E-cadherin and p38 MAPK and ERK1/2 and the phosphorylated (p-)forms of the kinases in Huh7 and HepG2 cells. (**A**) p38 and p-p38 MAPK in Huh7 cells overexpressing chemerin isoforms. (**B**) Ratio of p-p38 to p38 MAPK in these cells. (**C**) ERK1/2 and p-ERK1/2 in Huh7 cells overexpressing chemerin isoforms. Bands are from a single image and were sorted in the same order as the other immunoblots shown. (**D**) Ratio of p-ERK1/2 to ERK1/2 in these cells. (**E**) p38 and p-p38 MAPK in HepG2 cells overexpressing chemerin isoforms. (**F**) Ratio of p-p38 to p38 MAPK in these cells. (**G**) ERK1/2 and p-ERK1/2 in HepG2 cells overexpressing chemerin isoforms. (**H**) Ratio of p-ERK1/2 to ERK1/2 in these cells. (**I**) E-cadherin in Huh-7 cells overexpressing chemerin isoforms. (**J**) Quantification of E-cadherin in Huh7 cells. (**K**) E-cadherin in HepG2 cells overexpressing chemerin isoforms. (**L**) Quantification of E-cadherin in HepG2 cells. Data were analyzed with one-way ANOVA with post-hoc Tukey test or the Welch test with post-hoc Games–Howell test; *n* = 3–4.

Chemerin regulates ERK1/2 and p38 MAPK activities, which are central pathways in tumor progression and metastasis [5,6,8]. The effect of chemerin isoform overexpression on the activation of these proteins was investigated. Phosphorylated forms of ERK1/2 and p38 MAPK were analyzed by immunoblot in all three cells lines, but there was no effect of any chemerin isoform (Figure 6A–H and Supplementary Figure S1A,B).

Epithelial–mesenchymal transition (EMT) is crucial for cancerogenesis and downregulation of E-cadherin is a characteristic of this process [24]. E-cadherin protein was, however, not regulated by any chemerin isoform (Figure 6I–L and Supplementary Figure S1C).

### 2.7. Alpha-Fetoprotein and Cytokines in the Cell Culture Media of Chemerin Isoform-Overexpressing Hepatocytes

Paracrine and autocrine signals contribute to migration of hepatocytes. These factors may be suppressed by the highly active chemerin isoforms. Moreover, abundance of these molecules may vary between HepG2 and Huh7 cells and explain the lack of an effect on cell migration in the Huh7 cell line (Figure 5).

**Figure 7:**
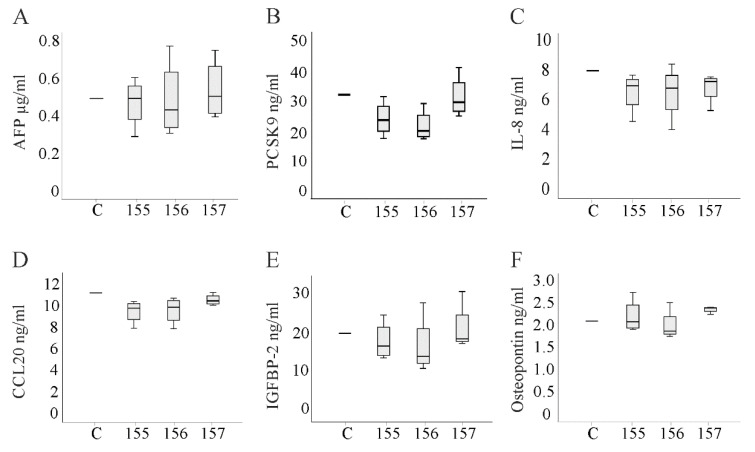
Proteins measured in the media of transfected Huh7 cells by ELISA. (**A**) Alpha-fetoprotein (AFP); (**B**) proprotein convertase subtilisin/kexin type 9 (PCSK9); (**C**) IL-8; (**D**) CCL20; (**E**) insulin-like growth factor-binding protein-2 (IGFBP-2); (**F**) osteopontin. n = 4. Data were analyzed with one-way ANOVA with post-hoc Tukey test or the Welch test with post-hoc Games–Howell test.

Alpha-fetoprotein (AFP) is a widely used HCC marker [25] and increases cell migration [26]. However, levels in the media of the three cell lines did not vary (the median concentration of AFP in media of control-transfected HepG2 cells was 7.9in Huh7 cell media was 4.8 and in Hepa1-6 cell media was 3.1 µg/mL / per 1 million cells (*n* = 4)). Moreover, none of the chemerin isoforms regulated AFP in the cell lines (Figure 7A and Supplementary Figure S2A,B).

Hybridization of a human cytokine array with media of HepG2 and Huh7 cells revealed that trefoil factor 3 and vascular endothelial growth factor were abundant in HepG2 but not Huh7 cells (Table 1 and Supplementary Figure S3). These factors induce migration of hepatocytes [27,28] but were not diminished upon huChem-157 overexpression (Supplementary Figure S3).

The medium of Hepa1-6 cells was used for hybridization of a murine cytokine array (Table 1 and Supplementary Figure S3). Trefoil factor 3 antibodies were not spotted on the membranes and vascular endothelial growth factor was not regulated by muChem-156 (Supplementary Figure S3). AFP and vascular endothelial growth factor were not downregulated by huChem-157 or muChem-156, and most likely do not have a role in reduced migration of HepG2 and Hepa1-6 cells (Figure 5B,C,E,F).

Overall, the cytokine array experiments showed that Hepa1-6 cells produced more different cytokines than the human cells (Supplementary Figure S3). Thirty-seven proteins were detected either in the media of murine or human hepatocytes, and 21 proteins were identified by both arrays. Of these 21 proteins, 13 differed between Hepa1-6 cells and Huh7 cells, 11 between Hepa1-6 cells and HepG2 cells and 4 between Huh7 cells and HepG2 cells (Table 1).

Crosstalk between HCC cells and immune cells involves cytokines produced by hepatocytes [22]. Chemerin isoforms did not greatly alter the levels of proteins in the cell media (Supplementary Figure S3). Quantification of the signals with ImageJ suggested a modest upregulation of CCL2 and IL-8 by huChem-157, a downregulation of osteopontin by huChem-156, suppression of CCL20 by huChem-157, and IGF-binding protein-2 by both active chemerin isoforms in Huh7 cells. Proprotein convertase 9 (PCSK9) was induced in Hepa1-6 cells by muChem-155 (Supplementary Figure S3). Analysis of PCSK9 (Huh7 and HepG2), IL-8 (Huh7 and HepG2 and the murine homolog KC in Hepa1-6 media), CCL20 (Huh7 and HepG2), IGF-binding protein-2 (Huh7 and HepG2) and osteopontin (Huh7 and HepG2) in the cell culture media by ELISA could not identify any factor that was significantly changed by a chemerin isoform. These results were shown for Huh7 cells (Figure 7B–F).

### 2.8. Cytokines of PBMCs Cultivated in Hepatocyte-Conditioned Medium

Hepatocyte-released chemerin or other so far unknown soluble factors secreted by chemerin-overexpressing cells may have an impact on immune cells. Therefore, human PBMCs were cultivated for 24 h in conditioned media of Huh7 cells. CCL2 and IL-8 act as chemoattractants, IL-6 is the major cytokine in acute phase response and osteopontin is a central mediator of liver fibrosis [29,30]. CCL20 is a tumor-promoting chemokine, and induces EMT and cell migration [31]. Tumor necrosis factor (TNF) is a well-studied inflammatory cytokine and can promote or block tumor progression [32]. The function of these proteins in cancer is well established [29,30,31,32] and their levels were measured by ELISA in media of PBMCs.

**Figure 8:**
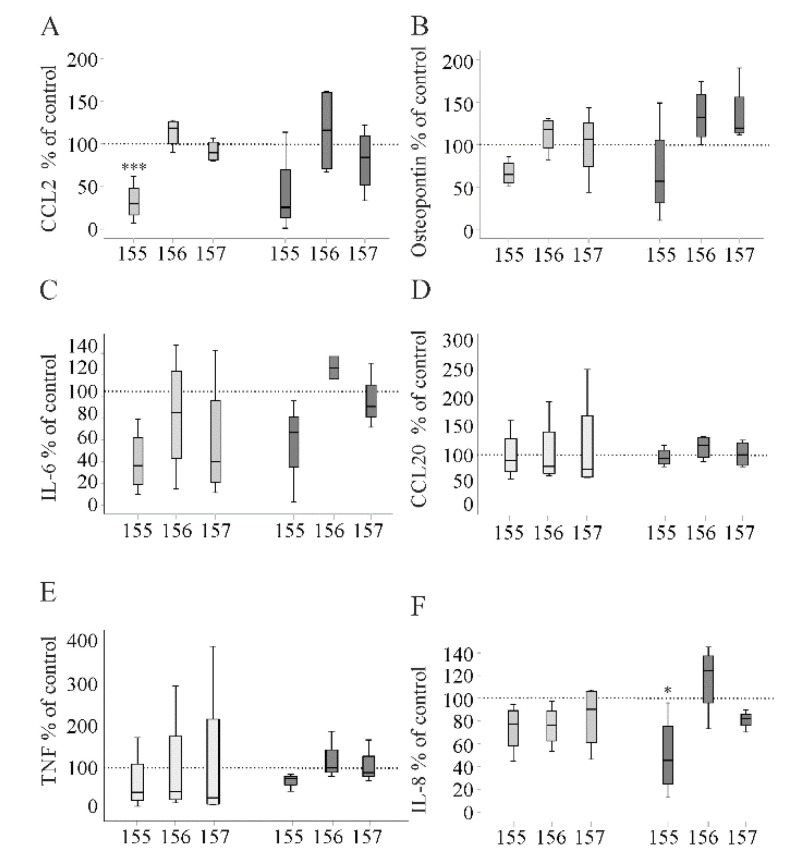
Proteins measured in the media of peripheral blood mononuclear cells (PBMCs) cultivated in Huh7 cell-conditioned medium by ELISA. (**A**) CCL2; (**B**) osteopontin; (**C**) IL-6; (**D**) CCL20; (**E**) TNF; (**F**) IL-8. Light gray boxes are cells without and dark gray boxes are cells with 100 ng/ml lipopolysaccharide in the cell media. Data were analyzed with one-way ANOVA with post-hoc Tukey test or the Welch test with post-hoc Games–Howell test. * *p* < 0.05; *** *p* < 0.001; compared to control-cultivated PBMC, which were set to 100% (dotted line), *n* = 4.

The hepatocyte-conditioned medium of huChem-155 producing Huh7 cells reduced IL-6, osteopontin and CCL2, of which the latter effect was significant (Figure 8A–C). CCL20 and TNF were not regulated by the chemerin isoforms (Figure 8D,E). There was a marginal suppressive effect of huChem-155 and 156 on soluble IL-8 levels (Figure 8F).

Supplementation of the conditioned culture medium with lipopolysaccharide (100 ng/mL) did not result in higher levels of IL-6 (Figure 8C). The transfection reagent lipofectamine was described to inhibit the pro-inflammatory response of macrophages upon LPS stimulation [33]. Similar to non-LPS-treated PBMCs, huChem-155 lowered levels of CCL2, osteopontin, IL-6 and IL-8, and, in this experimental setting, the suppressive effect on IL-8 was significant (Figure 8A–C,F). A marginal suppressive effect on TNF was seen in huChem-155-incubated PBMCs (Figure 8E). Again, CCL20 levels were not regulated by recombinant chemerin variants (Figure 8D).

Here, it is important to note that CCL2, TNF and IL-6 were hardly detectable in Huh7 cell media. IL-8 was approximately 5- to 10-fold higher in PBMCs than Huh7 cell media. Osteopontin and CCL20 levels were comparable in Huh7 and PBMC media. This suggests that IL-6, IL-8, CCL2 and TNF measured in PBMC media were mostly released by the immune cells. Regarding osteopontin and CCL20, the cellular origin is less clear.

## 3. Discussion

Current experiments provide evidence that human HCC cells overexpressing huChem-155 exert anti-inflammatory effects in PBMCs. HuChem-157 and muChem-156 inhibit migration of HepG2 and Hepa1-6 cells, respectively. This suppressive activity was not observed in the Huh7 cell line.

The Huh7 cells detached from the plate at 72 h post-transfection and for this cell line, analysis was performed at 48 h. This is a limitation of the scratch assay regarding Huh7 cells and Transwell assays may be better suited to study migration of these cells.

In the present analysis, three different HCC cell lines were used—one murine and two human. HCC is a heterogenous malignancy and diverse genomic aberrations were discovered in HCC cell lines [34]. Thus, HepG2 and Hepa1-6 cells express normal p53, and Huh7 a mutated p53 protein [35,36,37]. Moreover, the p53 gene regulatory network is highly divergent between mice and humans [38]. Analysis of soluble proteins in HCC cell culture media also revealed differences between the murine and human cell lines. Approximately 50% of the proteins, which could be detected by the human and mouse cytokine arrays, differed between the murine and human HCC cells. Furthermore, CCL2, CCL5, CCL17 and GM-CSF for instance were detected only in the cell culture medium of the murine cell line. A recent study showed that muChem-156-overexpressing Hepa1-6 cells produced lower levels of GM-CSF [8]. Such a downregulation was not observed in the Hepa1-6 cells overexpressing muChem-156 in the present study. There was approximately 200 ng/mL of muChem-156 in the present analysis and approximately 350 pg/mL in the recent study [8]. Dose-response curves are needed to analyze a potential bell-shaped effect of chemerin on GM-CSF production. Moreover, GM-CSF could not be detected in the cell culture medium of the human cells. GM-CSF is a potent immune-stimulatory factor and may be used as an adjuvant in tumor therapy [39]. Thus, the regulation of GM-CSF by chemerin warrants further study.

Four proteins varied between HepG2 and Huh7 cells. Huh7 cells produced CXCL5 and fibroblast growth factor 19 at a concentration that was detectable by the array, whereas trefoil factor 3 and vascular endothelial growth factor were only found in the media of HepG2 cells. Mutant p53 is expressed in Huh7 cells and was shown to induce CXCL5, which contributes to HCC cell proliferation [35,40,41]. Fibroblast growth factor 19 was not detected in the HepG2 cell culture medium by mass spectrometry [42] in accordance with current data using a cytokine array. Vascular endothelial growth factor and fibroblast growth factor 19 DNA amplifications were detected in more than 5% of HCC patients [43] and may explain higher levels in the Huh7 cell line. Trefoil factor 3 is upregulated in HCC tissues and contributes to HCC cell proliferation and migration [27]. HuChem-157 did not reduce the levels of trefoil factor 3 or vascular endothelial growth factor in cell media. Differential abundance of these two proteins in the media of the human cell lines most likely cannot explain why huChem-157 lowered migration of HepG2 but not Huh7 cells.

Chemerin was hardly expressed in the three HCC cell lines. Overexpression of chemerin isoforms did not grossly change the levels of different proteins in the culture media of the hepatocytes. This was verified for AFP, IL-8, CCL20, IGFBP-2, osteopontin and PCSK9 by ELISA. IL8 and CCL20 promoted migration and invasion of HCC cells [44]. IGFBP-2 and osteopontin contributed to HCC cell proliferation and migration [30,45]. Low PCSK9 in HCC is known to enable a constant cholesterol supply, which is essential for cell growth [46].

IL-8 and osteopontin were not regulated by huChem-157 in PBMC. The protective activity of muChem-156 in HCC, which resembles huChem157 [1,6,8,9], appears not to impact these molecules. Moreover, central pathways in cell proliferation and migration [5,6,8] were not activated in hepatocytes with chemerin isoform overexpression. Levels of phosphorylated p38 and ERK1/2 were not regulated by any chemerin variant. Both of these pathways were regulated by chemerin in tumor cells [5]. Dose-dependent effects of chemerin on the activation of ERK1/2 and AKT were described, and low, but not high, levels of recombinant chemerin induced phosphorylation of these proteins [1,6]. These finding do not exclude that MAPK kinases were activated by chemerin overexpression, e.g., early after transfection, where chemerin levels are low.

Transforming growth factor-β activates p38 MAPK and ERK and contributes to tumor progression [47]. Loss of E-cadherin is a hallmark of EMT, and is linked to activation of TGF-β signaling [24]. E-cadherin was not regulated by any chemerin isoform, and p38 MAPK and ERK1/2 were not activated, suggesting that chemerin did not regulate EMT. Biological activity of recombinant chemerin was verified by the Tango assay. This analysis confirmed that huChem-157 overexpressed in HepG2 and Huh7 cells was the most effective isoform at activating CMKLR1 and GPR1. HuChem-156 derived from HepG2 cells was a less active ligand for CMKLR1. When expressed in Huh7 cells, huChem-156 did not activate either of the chemerin receptors. The concentration of huChem-156 in the Huh7 cell culture medium was approximately 3-fold lower in comparison to HepG2 cells and thus this low activation of CMKLR1 and GPR1 was not significant.

MuChem-155 was as effective as muChem-156 in activating human GPR1 and murine CMKLR1. Zhao et al. reported a comparable chemotactic activity of these isoforms on muCMKLR1/L1.2 cells [12]. The ability of muChem-155 to activate huCMKLR1 was much lower in comparison to muChem-156. This illustrates that human, but not murine CMKLR1 differentially responded at least to the murine chemerin isoforms. The lower activity of huChem-156 versus huChem-157 may be a specific feature of the human system and evaluation of the molecular mechanisms may further contribute to a better understanding of CMKLR1 signaling pathways.

Mass spectrometric analysis of recombinant chemerin isoforms was performed to confirm the identity of the recombinant chemerin proteins. MuChem-155 and -156 were the only isoforms identified in the cell culture media of Hepa1-6 cells transfected with the corresponding plasmids. MuChem-154 overexpression resulted in muChem-154 and -153 illustrating further processing of this chemerin isoform. C-terminal shortening of muChem-154 was not observed in CHO-S cells which, however, partly processed muChem-157 to muChem-154 [12]. In Huh7 cells producing huChem-157, the isoforms huChem-157, -156, -155 and -154 were detected. Huh7 cells, however, did not further process huChem-156. Altogether, these findings show that processing of chemerin isoforms differs in murine and human HCC cells

HuChem-157 expressed in HepG2 cells and muChem-156 derived from Hepa1-6 cells both efficiently reduced cell migration which is in line with a previous study [6]. Inhibition of migration was not achieved by any other isoform, which suggests that this activity is specific for huChem-157/muChem-156. Processing of huChem-157 in Huh7 cells to shorter isoforms most likely prevented inhibition of cell migration in this cell line. Recruitment of beta-arrestin 2 to human GPR1 and murine CMKLR1 by muChem155 and muChem-156 was comparable, illustrating that both isoforms are effective ligands for these receptors. The inhibitory activity of chemerin in cell migration involves weakening of the PTEN–CMKLR1 interaction and subsequent accumulation of PTEN in the cells [6]. This activity of chemerin may not include beta-arrestin 2 downstream pathways. MuChem-156 but not muChem-155 seems to be able to disrupt the CMKLR1–PTEN complex.

As expected, muChem-154 and huChem-155 did not activate CMKLR1 or GPR1 in the respective Tango assays [1]. Surprisingly, overexpression of huChem-155 in Huh7 cells reduced IL-6, IL-8, osteopontin and CCL2 in the culture media of PBMCs. A marginal suppressive effect on TNF was also noted. An effect on these molecules was not observed for the other chemerin isoforms. Moreover, chemerin activity was not detected in the control-transfected cells. Therefore, it is unlikely that huChem-155 simply blocked the active chemerin isoforms. Rather, this suggests that huChem-155 may activate a chemerin receptor signaling mechanism that is independent of beta-arrestin-2 recruitment, which is the pathway tested by the Tango assay. In addition to beta-arrestin 2, binding of chemerin also induced beta-arrestin 1 recruitment to CMKLR1 and GPR1 [19] and further research is needed to clarify the signaling pathways activated by this short isoform. Future experiments are also needed to determine whether huChem-155 directly modifies the PBMC secretome.

## 4. Materials and Methods

### 4.1. Cell lines and Primary Cells

HepG2 cells, Huh7 cells and Hepa1-6 cells were from the German Collection of Microorganisms and Cell Cultures GmbH (Braunschweig, Germany). HepG2 cells were cultivated in RPMI medium supplemented with 10% fetal calf serum and 1% penicillin/streptomycin. Huh7 and Hepa1-6 cells were cultivated in DMEM medium supplemented with 10% fetal calf serum and 1% penicillin/streptomycin. Cell number was determined by the Countess II FL from Life Technologies (Thermo Fisher Scientific, Waltham, MA USA). Here, live and dead cells are discriminated. The advantage of this methodology is that the subjectivity of manual counting is eliminated and that the user-to-user variability is low.

Peripheral blood mononuclear cells (PBMCs) of four donors were ordered from Hepacult GmbH (Regensburg, Germany). PBMCs were cultivated in RPMI medium supplemented with 10% fetal calf serum and 1% penicillin/streptomycin. The medium was removed and PBMCs were cultivated in the conditioned medium of hepatocytes for 24 h. Lactate dehydrogenase (LDH) in the cell culture medium was measured by the Cytotoxicity Detection Kit from Roche (Mannheim, Germany).

### 4.2. Expression of Recombinant Human Chemerin Isoforms

Polymerase chain reaction to amplify human chemerin cDNA employed the universal primer 5’- CGA AAG CTT ATG CGA CGG CTG CTG ATC C -3’ and the reverse primers 1) huChem-157: 5´- CGA CCG CGG TTA GGA GAA GGC GAA CTG TCC AGG -3 2) huChem-156: 5´- CGA CCG CGG  TTA GAA GGC GAA CTG TCC AGG GAA-3 and 3) 5´- huChem-155 CGA CCG CGG  TTA GGC GAA CTG TCC AGG GAA GTA-3. For cloning of murine chemerin isoforms, the universal primer 5´- CGA AAG CTT ATG AAG TGC TTG CTG ATC TCC CTA -3’ and the reverse primers 1) muChem-156: 5´- CGA CCG CGG TTA GGA GAA GGC AAA CTG TCC AGG -3 2) muChem-155: 5´- CGA CCG CGG TTA GAA GGC AAA CTG TCC AGG TAG-3 and 3) muChem-154: 5´- CGA CCG CGG TTA GGC AAA CTG TCC AGG TAG GAA-3´ were used. The cutting sites for the restriction enzymes HindIII and SacII are underlined. The DNA was cloned in the vector pcDNA3.1 (Thermo Fisher Scientific, Waltham, MA, USA). The DNA sequences of the fragments were verified by sequence analysis (GeneArt ThermoFisher, Regensburg, Germany). Transfection of cells was performed with Lipofectamine^TM^ 3000 Reagent (Thermo Fisher Scientific).

### 4.3. SDS-PAGE and Immunoblotting

Proteins (20 µg) were separated by SDS-polyacrylamide gel electrophoresis and blotted to PVDF membranes (Bio-Rad, Munich, Germany). Membranes were incubated with antibodies in 1.5% BSA/PBS/0.1% Tween 20. Signals were detected with the ECL Western blot detection system (Amersham Pharmacia, Deisenhofen, Germany). E-cadherin, ERK1/2, GAPDH and p38 MAPK antibodies were from New England Biolabs GmbH (Frankfurt, Germany). Antibodies to detect human and murine chemerin were from R&D Systems (Wiesbaden-Nordenstadt, Germany). ImageJ software was used for quantification [48].

### 4.4. ELISAs, MTT Assay and Cytokine Array

ELISAs were ordered from R&D Systems and performed as recommended by the company. The MTT Cell Proliferation and Cytotoxicity Assay Kit was from Boster Immunoleader (Cologne, Germany). Proteome Profiler^™^ Human / Mouse XL cytokine arrays were from R&D Systems and were hybridized with the cell culture medium as recommended by the company.

### 4.5. Tango Assay

Chemerin activation of CMKLR1 and GPR1 was determined by the Tango assay as described in detail [11,49].

### 4.6. Scratch Assay

For wound-healing migration assay, cells were seeded on 24-well plates. A scratch was generated immediately after transfection of the cells with the recombinant plasmids. Images were captured at 48 after scratching.

### 4.7. Mass Spectrometry of Chemerin Isoforms

Chemerin was precipitated from the cell culture medium by the Pierce^TM^ Classic Magnetic IP/CoIP kit (Thermo Fisher Scientific, Waltham, MA, USA) and the chemerin antibody AF2324 for human and AF2325 for the murine protein (R&D Systems). Mass spectrometry of chemerin protein was described recently in detail [9].

### 4.8. Statistical Analysis

Statistical analysis was performed using the mean values of the technical replicates. Data are presented as box plots. Statistical differences were analyzed by ANOVA with post-hoc Tukey, Welch test with post-hoc Games–Howell or Mann–Whitney U test, and a value of *p* < 0.05 was regarded as significant (SPSS Statistics 25.0 program, IBM, Leibniz Rechenzentrum, München. Germany).

## 5. Conclusions

The present study showed that chemerin signaling is highly complex. HuChem-157 inhibited cell migration and huChem-155 exerted anti-inflammatory activity in immune cells. Chemerin isoforms thus act through different mechanisms to protect from HCC. A combination of chemerin isoforms or the respective biologically active C-terminal peptides may be a highly efficient anti-HCC therapy.

## Figures and Tables

**Table 1:** Proteins which were detected in the media of murine and/or human hepatocytes by the respective antibody arrays are listed. A total of 37 proteins were found expressed, and 21 were detectable by the murine and the human array. The n.i. symbol indicates that antibodies to this protein were not included in this array. The proteins produced by the respective cell lines are marked with x. Proteins which were not detected in the media are marked with n. d. (not detected). Proteins produced by either Huh7 or HepG2 cells are in bold.

Protein	Hepa1-6	Huh7	HepG2
Amphiregulin	x	-	-
Angiogenin	-	x	x
Apolipoprotein A1	-	x	x
CCL2/Monocyte chemoattractant protein-1	x	n.d.	n.d.
CCL5/Regulated upon activation, normal T cell expressed, and secreted	x	n.d.	n.d.
CCL17/Thymus and activation regulated chemokine	x	n.d.	n.d.
CCL20/Macrophage inflammatory protein-3	x	x	x
CXCL1/KC/IL-8	x	x	x
CXCL2/Macrophage inflammatory protein-2	x	-	-
**CXCL5/E** **pithelial-derived neutrophil-activating peptide 78**	x	x	n.d.
CXCL10/Interferon gamma-induced protein 10	x	n.d.	n.d.
CXCL16	x	-	-
Cystatin C	x	x	x
Dickkopf-1	n.d.	x	x
Dipeptidylpeptidase	n.d.	x	x
Extracellular matrix metalloproteinase inducer	n.i.	x	x
Endostatin	x	n.i.	n.i.
**Fibroblast growth factor-19**	n.i.	x	n.d.
Growth differentiation factor-15	x	x	x
Granulocyte-macrophage colony-stimulating factor	x	n.d.	n.d.
Intercellular adhesion molecule 1	x	n.d.	n.d.
IGF-binding protein (IGFBP)-1	x	n.i.	n.i.
IGFBP-2	n.d.	x	x
IGFBP-3	x	n.d.	n.d.
IL-23	x	n.d.	n.d.
Macrophage inflammatory protein	n.i.	x	x
Matrix metalloproteinase-3	x	n.i.	n.i.
Osteopontin (OPN)	x	x	x
Osteoprotegerin/TNFRSF11B	x	n.i.	n.i.
Pentraxin 3	n.d.	x	x
Proprotein convertase subtilisin/kexin type 9/PCSK9	x	n.i.	n.i.
Retinol-binding protein 4	x	x	x
Serpin E1/Plasminogen activator inhibitor-1	x	x	x
Thrombospondin-1	n.i.	x	x
**Trefoil factor 3**	n.i.	n.d.	x
Tissue factor	x	n.i.	n.i.
**Vascular endothelial growth factor**	x	n.d.	x

## Supplementary Materials

Supplementary materials can be found at https://www.mdpi.com/1422-0067/21/19/7205/s1.
