# Supplementary material for: Chemerin Isoform-Specific Effects on Hepatocyte Migration and Immune Cell Inflammation
Source: Int J Mol Sci. 2020 Sep 29;21(19):7205. doi: 10.3390/ijms21197205 (PMC7582997; doi:10.3390/ijms21197205)
Supplement: Supplementary file 1 [file ijms-21-07205-s001.pdf]

Supplementary Table 1. Optical density values of lactate dehydrogenase in cell media of Hepa1-6, HepG2 and Huh7 cells 48 h post-transfection (n = 12 -13).

LDH	Isoform	Hepa1-6	Isoform	HepG2	Huh7
Median	Control	0.3	Control	0.3	0.4
Minimum		0.2		0.1	0.3
Maximum		0.6		0.5	0.7
Median	154	0.3	155	0.3	0.5
Minimum		0.2		0.1	0.2
Maximum		0.6		0.5	0.7
Median	155	0.3	156	0.3	0.5
Minimum		0.2		0.1	0.3
Maximum		0.6		0.5	0.7
Median	156	0.3	157	0.3	0.4
Minimum		0.2		0.1	0.3
Maximum		0.6		0.5	0.6

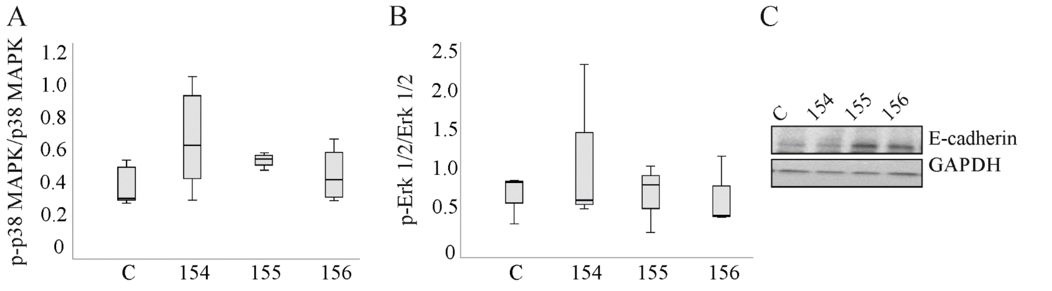

Supplementary Figure S1. Expression of E-cadherin and p38 MAPK and ERK1/2 and the phosphorylated (p-)forms of the kinases in Hepa1-6 cells. (**A**) Ratio of p-p38 to p38 MAPK in Hepa1-6 cells overexpressing chemerin isoforms. (**B**) Ratio of p-ERK1/2 to ERK1/2 in these cells. (**C**) E-cadherin in Hepa1-6 cells overexpressing chemerin isoforms. Data were analyzed with one-way ANOVA with post-hoc Tukey test or the Welch test with post-hoc Games–Howell test; *n* = 3–5.

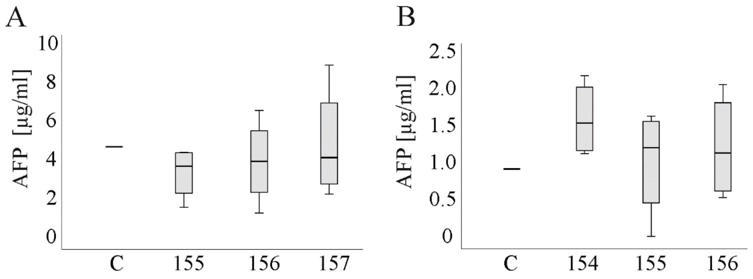

Supplementary Figure S2. Alpha-fetoprotein (AFP) measured in the media of transfected HepG2 and Hepa1-6 cells by ELISA. (**A**) HepG2 cells; (**B**) Hepa1-6 cells. n = 4 - 5. Data were analyzed with one-way ANOVA with post-hoc Tukey test.

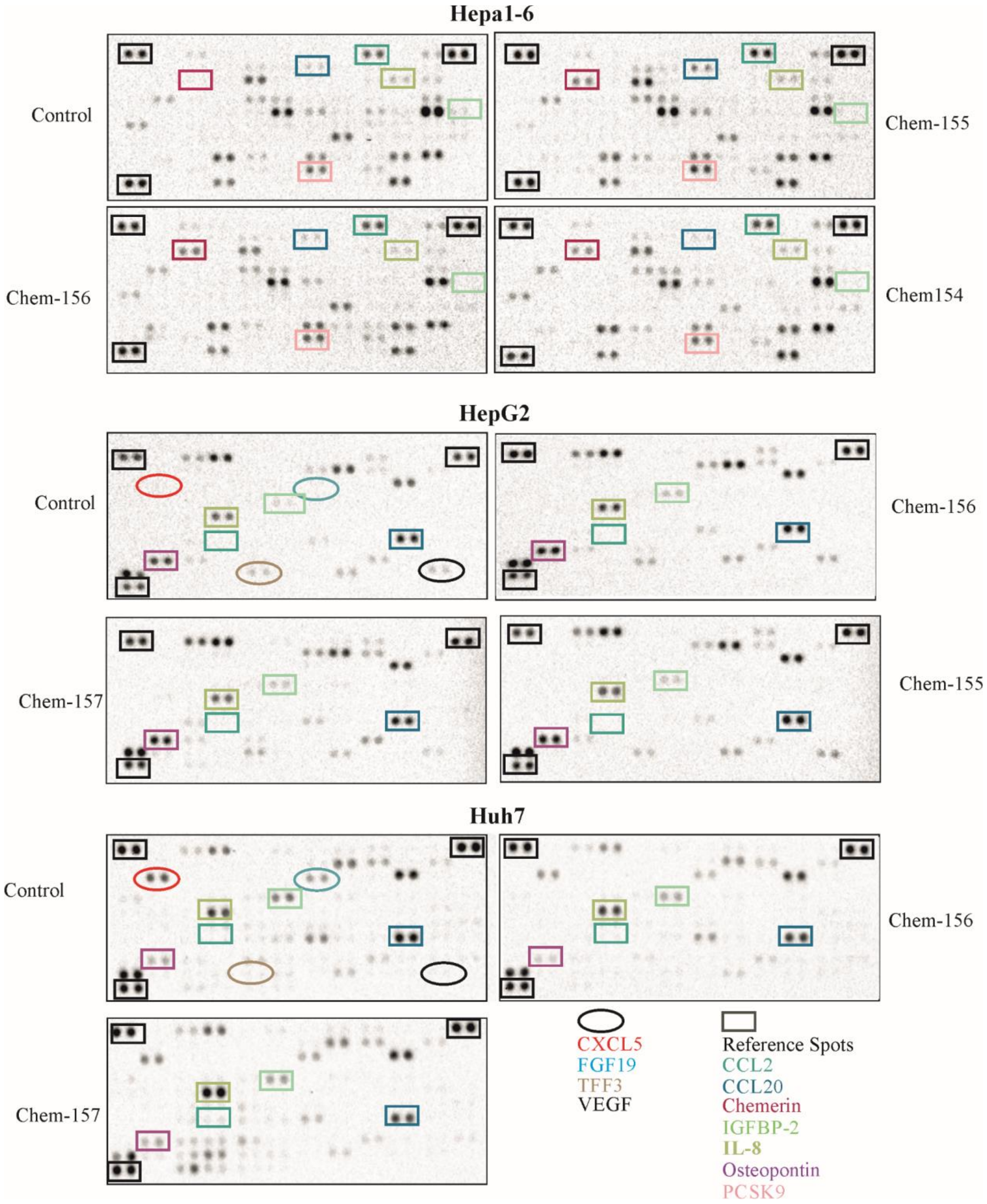

Supplementary Figure S3. Cytokine Arrays. Cytokine Arrays were hybridzed with media of cells overexpressing chemerin isoforms or cells transfected with an insertless plasmid (Control). Proteins marked by ellipses were differentially abundant in the human cells compared to the murine cell line. Proteins marked by a box seem to be regulated by chemerin isoform overexpression. The protein names are given in the color of the respective symbols.
